# Laparoscopic ventral TAPP versus IPOM for primary umbilical hernia repair: why use a sledgehammer to crack a nut?

**DOI:** 10.3389/jaws.2026.16805

**Published:** 2026-07-30

**Authors:** Dimitrios Prassas, Omar Kreym, Wolfram Trudo Knoefel, Sascha Vaghiri

**Affiliations:** 1 Department of General and Visceral Surgery, Katholisches Klinikum Essen, Teaching Hospital of Duisburg-Essen University, Essen, Germany; 2 Heinrich Heine University of Düsseldorf and Düsseldorf University Hospital, Department of Surgery (A), Düsseldorf, Germany

**Keywords:** IPOM, IPOM PLUS, umbilical hernia, ventral hernia, ventral TAPP

## Abstract

**Introduction:**

Intraperitoneal onlay mesh (IPOM) repair is the most commonly performed minimally invasive technique for ventral hernia repair, however, it is associated with increased postoperative discomfort and longer hospitalization. This study aimed to compare the efficacy and safety of the ventral transabdominal preperitoneal (V-TAPP) procedure and IPOM in primary umbilical hernia repair.

**Patients and methods:**

A retrospective review was conducted on 70 adult patients who underwent elective laparoscopic primary umbilical hernia repair between 2019 and 2026, using either the V-TAPP (n = 40) or IPOM (n = 30) technique. Short- and long-term comparative outcome analyses were performed.

**Results:**

Patient demographics, comorbidities and hernia characteristics were similar between the two groups. Surgery duration was significantly shorter in the IPOM group (time in minutes V-TAPP vs. IPOM: 98.1 ± 26.3 vs. 68.4 ± 24.5; p < 0.001). The effect of IPOM with regard to postoperative pain at 24 h was also pronounced (VAS Score min:0 max:10; V-TAPP vs. IPOM: 3 ± 1.3 vs. 4.8 ± 1.4; p < 0.001) Postoperative LOS was significantly longer in the IPOM group (LOS in days V-TAPP vs. IPOM: 1.3 ± 1 vs. 4.8 ± 1.4; p < 0.001). After adjusting for baseline clinical confounders via multivariable regression, the IPOM approach remained a significant independent predictor of both longer hospital stays (B = 2.69, p < 0.05) and higher 24-h postoperative pain (B = 1.81, p < 0.05). All other intra- and perioperative outcomes of interest were similar. No differences were noted at follow-up.

**Conclusion:**

With the exception of a longer operative time, V-TAPP was associated with significant advantages over IPOM in terms of patient recovery, while maintaining comparable safety and efficiency. While these findings are promising, larger prospective studies are warranted to confirm whether V-TAPP should be considered the preferred approach for small- and medium-sized primary umbilical hernias.

## Introduction

Ventral hernia repair is one of the most frequently performed procedures worldwide with 300.000 cases being performed annually in Europe alone [1]. Laparoendoscopic techniques are well established as appropriate treatment options for small-to medium-sized ventral hernias, and their use has been incorporated into international guidelines [2]. Despite this, no consensus has been reached with regard to the most appropriate anatomical position of the mesh. Since its introduction, nearly two and a half decades ago [3], laparoscopic intraperitoneal onlay mesh repair (IPOM) has gained remarkable acceptance as an efficient and easily-reproducible method. Its obvious inherent drawbacks are associated with the onlay positioning itself. The mesh has direct contact to the abdominal viscera and needs to be fixated causing, in spite of the minimally invasive nature of the procedure, comparable pain to the open approach and potentially intraperitoneal postoperative adhesions [4]. Both of the above-mentioned factors are eliminated if the mesh is positioned in the extraperitoneal space, as in the ventral trans-abdominal pre-peritoneal (V-TAPP) approach. Moreover, because the mesh does not come into contact with the intraperitoneal cavity, a protective coating is unnecessary, making the V-TAPP approach more cost-effective [5]. As an attempt to compare the short- and long-term efficacy and safety of the two above mentioned methods, we conducted a retrospective single-center cohort analysis.

### Patients and methods

#### Objectives and study design

Between January 2019 and February 2026, a total of 351 patients underwent elective ventral hernia repair at our institution, of whom 195 had umbilical hernias. Seventy of these were operated on laparoscopically, either using the laparoscopic IPOM (n = 30) or laparoscopic V-TAPP (n = 40) approach. Patients were asked to assess pain severity using a ten-point scale preoperatively, 24 h postoperatively, and at follow-up. Follow-up was conducted during outpatient visits, where the operating surgeons evaluated patients clinically and sonographically for postoperative seroma, hernia recurrence, and chronic pain. After discharge, patients were scheduled for follow-up at 30 days and approximately 1 year postoperatively. The minimum follow-up period for long-term clinical assessment was 2 months postoperatively.

Medical and operative records for each included patient were reviewed to collect the following parameters: 1) demographics [age, sex, and body mass index (BMI)], American Society of Anesthesiologists (ASA) classification, and comorbidities; 2) operative details [type of surgery, duration of surgery (min), conversion to another surgical approach, and hernia size]; and 3) postoperative outcomes [hematoseroma, intraperitoneal hematoma, surgical site infection/mesh infection], overall morbidity, and postoperative hospital stay (LOS).

Outcomes of interest included intraoperative (surgery duration, intraoperative complications, conversion rate), short-term postoperative (overall morbidity, pain level at 25 h postoperatively, seroma rate at 30 days) and long-term follow-up outcomes (recurrence, chronic pain).

#### Surgical technique

All procedures were performed by five surgeons with expertise in advanced laparoscopic surgery. In October 2023, the V-TAPP approach was introduced at our institution by the corresponding author (DP) and subsequently established as the standard technique for small-to medium-sized umbilical hernias. All operating surgeons were beyond their learning curve for the included analysed cases. The IPOM repair was performed in three trocar technique by placing a coated monofilament polyester mesh (Symbotex™ composite mesh, Medtronic) with 5 cm overlapping borders of the hernia defect, after closure of the defect with 1/0 Vicryl. The mesh was then positioned intraperitoneally with two transfascial sutures. Additional fixation with absorbable tacks in double-crown technique was performed. The V-TAPP procedures were also performed using a three-trocar technique. The first step, after establishing capnoperitoneum, is insufflation with a Veress needle introduced at Palmer’s point into the extraperitoneal space. This maneuver facilitates easier dissection of the preperitoneal space and, in individuals with lower BMI, the needle itself can be used as a dissecting instrument, enhancing the effect of pneumodissection.

Subsequently, the peritoneum is grasped with a Maryland dissector and incised with scissors approximately 6 cm lateral to the hernia defect. This is the most technically demanding step of the procedure and can be facilitated by the assistant applying external pressure to the abdominal wall against the pneumoperitoneum. After the initial incision, it is extended craniocaudally, parallel to the midline, over a distance of approximately 15 cm, depending on the size of the hernia.

The extraperitoneal space is then entered and developed toward the midline, taking care to avoid peritoneal tears. Although this step is demanding, it becomes easier once the preperitoneal fat pad at the midline is reached. The space is subsequently expanded to the contralateral side, both cranial and caudal to the hernia sac. Reduction of the hernia sac is performed only after the peritoneum has been circumferentially dissected from the hernia ring, creating the so-called “volcano sign.”

The hernia sac is then reduced, and the peritoneal flap is extended to the opposite side in a mirror-image fashion to allow placement of a mesh with at least a 5 cm overlap beyond the defect margins. Any inadvert peritoneal defects can be closed with sutures or clips. The hernia defect is then closed using Vicryl 1/0, and a monofilament macroporous polypropylene mesh (Parietene™, Medtronic) is placed in the extraperitoneal space and secured with two transfascial sutures at the upper and lower midline. This constitutes the only mesh fixation. Finally, the peritoneal incision is closed with a continuous absorbable monofilament suture (PDS 2/0).

### Statistical analysis

Continuous variables were reported as mean ± standard deviation (SD) and assessed for normality prior to comparison. For normally distributed data, the t-test was applied, while the Mann-Whitney U test was used for non-normally distributed data. Categorical variables were expressed as frequencies (%) and compared using Fisher’s exact test or the chi-squared test as appropriate. Significance level was set at p ≤ 0.05. The analysis was performed on an intention-to-treat basis. To assess the robustness of our primary findings, multivariable linear regression analyses were conducted for the primary outcomes that demonstrated significant differences at baseline. Statistical analyses were conducted using SPSS software version 23.0 (IBM Corp., Armonk, NY).

### Ethics

The study was approved by the ethics committee of the Medical Faculty of Heinrich-Heine University of Düsseldorf (Study Nr. 2024-3013) and was conducted in compliance with the Strengthening the Reporting of Observational Studies in Epidemiology (STROBE) guidelines for observational research [6].

## Results

### Patient characteristics

The study included 70 cases of primary reducible umbilical hernias of small and medium size which underwent elective laparoscopic repair between January 2019 and February 2026. They were classified into two groups based on the surgical technique performed: the V-TAPP group (n = 40) and the IPOM group (n = 30). There were no statistically significant differences in demographic or hernia-associated characteristics. Baseline data of the study population are presented in Table 1.

**TABLE 1:** Demographics (ASA: American society of anesthesiologists classification, BMI: body mass index, COPD: chronic obstructive pulmonary disease, SD: stadard deviation, VAS: visual analogue score).

Baseline characteristics	V-TAPP (n = 40)	IPOM (n = 30)	*p*
Age±SD	55.5 ± 11.9	56.8 ± 14.3	0.667
Gender (M:F)	32:8	19:11	0.175
BMI±SD (kg/m^2^)	32.7 ± 6.8	33.3 ± 5.6	0.675
ASA	​	​	0.228
I	0	6.66% (n = 2)	​
II	72.5% (n = 29)	60% (n = 18)	​
III	27.5% (n = 11)	33.3% (n = 10)	​
Preop. VAS±SD (min:0; max:10)	2.4 ± 2.5	1.7 ± 1.7	0.265
Hernia size±SD (cm)	2.1 ± 0.4	2.3 ± 0.7	0.267
Risk factors	​	​	0.414
Smoking	7.5% (n = 3)	3.33% (n = 1)	​
COPD	5% (n = 2)	13.3% (n = 4)	​
Diabetes	10% (n = 4)	20% (n = 6)	​
Anticoagulation	7.5% (n = 3)	10% (n = 3)	​

### Outcomes

#### Intraoperative data

All procedures were performed laparoscopically with no case of conversion to open surgery. One case (2.5%) required conversion from V-TAPP to IPOM due to failure to raise a peritoneal flap. Intraoperative bleeding occurred in one case (3.3%) in the IPOM group and was related to lesion of an inferior epicastric artery by an absorbable tack. The complication was dealt with transfascial haemostatic stitches. Surgery duration was significantly shorter in the IPOM group (operative time in minutes V-TAPP vs. IPOM: 98.1 ± 26.3 vs. 68.4 ± 24.5; p < 0.001) (Tbl. 2).

#### Postoperative outcome

Overall postoperative morbidity was found to be similar in both groups [V-TAPP vs. IPOM: 7.5% (n = 3) vs. 6.6% (n = 2); p = 1] A more detailed description of postoperative morbidity is provided in Table 2. Return to the OR was deemed necessary for two cases, one in each group [V-TAPP vs. IPOM: 2.5% (n = 1) vs. 3.3% (n = 1); p = 0.677]. Patients in the V-TAPP group experienced significantly less pain 24h postoperatively (VAS Score min:0 max:10; V-TAPP vs. IPOM: 3 ± 1.3 vs. 4.8 ± 1.4; p < 0.001). Postoperative LOS was also significantly shorter in the V-TAPP group (LOS in days V-TAPP vs. IPOM: 1.3 ± 1 vs. 4.8 ± 1.4; p < 0.001). All cases were evaluated for seroma 30 days postoperatively. Two cases of asymptomatic seroma were noted, both in the V-TAPP group [V-TAPP vs. IPOM: 5% (n = 2) vs. 0; p = 0.503].

**TABLE 2:** Operative data and early postoperative outcome (LOS: length of stay SD: standard deviation, OR: operating room, VAS: visual analogue scale).

Intra- and perioperative outcome	V-TAPP (n = 40)	IPOM (n = 30)	p
Surgery duration±SD (minutes)	98.08 ± 26.32	68.37 ± 24.48	<0.001
Inraoperative morbidity	0	3.33% (n = 1)	0.429
Conversion	2.5% (n = 1)	0	1
Return to the operating room	2.5% (n = 1)	3.33% (n = 1)	0.677
Overall postoperative morbidity	7.5% (n = 3)	6.6% (n = 2)	1
Postoperative hematoseroma	5% (n = 2)	6.6% (n = 2)	1
Postoperative bleeding	2.5% (n = 1)	0	1
VAS at 24 h ± SD	2.98 ± 1.27	4.80 ± 1.40	<0.001
LOS ±SD (days)	1.28 ± 0.99	4.10 ± 2.28	<0.001
Seroma 30days postoperatively	5% (n = 2)	0	0.503
Mesh infection	0	0	-

To assess the robustness of our primary findings, multivariable linear regression analyses were conducted for the primary outcomes that demonstrated significant differences at baseline. After adjusting for potential clinical confounders—including age, BMI, ASA class, sex and hernia size, IPOM remained a significant independent predictor of both length of hospital stay (B = 2.69, p < 0.05 and postoperative pain at 24 h B= 1.81, p < 0.05.)

#### Follow-up

The follow-up rate for the V-TAPP group was 65% (n = 26), with a median follow-up of 13 months (range: 6–25), whereas the IPOM group had a follow-up rate of 86.6% (n = 26) and a median follow-up of 14.5 months (range: 2–82). The recurrence rate between the two procedures was not significantly different [V-TAPP vs. IPOM: 0% vs. 3.8% (n = 1); p = 1]. Chronic pain lasting over 3 months was noted in one case in the IPOM group [V-TAPP vs. IPOM: 0% vs. 3.8% (n = 1); p = 1]. No seromas were noted at follow up (Table 3).

**TABLE 3:** Follow-up.

Follow-up outcome	V-TAPP (n = 26)	IPOM (n = 26)	*p*
Mean follow-up in months (range)	13 (6–25)	14.5 (2–82)	0.004
Recurrence	0	3.8% (n = 1)	1
Chronic pain	0	3.8% (n = 1)	1
Seroma	0	0	-

## Discussion

More than three decades have passed since IPOM was first introduced [3]. It subsequently became the most commonly implemented technique before gradually losing its popularity by more contemporary extraperitoneal approaches [7]. This clear trend reflects the efforts of abdominal wall surgeons to develop a procedure that retains the efficacy of minimally invasive ventral hernia repair while avoiding the fundamental drawbacks of IPOM, namely mesh contact with the abdominal viscera and the need for mesh fixation. Intraperitoneal positioning of the mesh is regarded as a risk factor for adhesions and associated morbidity such as obstruction [8, 9]. With regard to peritoneal adhesions, Chelala et al. reported the outcomes of reoperations in patients with a history of IPOM, finding some degree of adhesion formation in approximately every second patient [10]. This led to the use of substantially more expensive coated meshes, which, however, do not fully prevent adhesion formation [11]. Anchoring of the mesh on the peritoneal surface is a critical step in IPOM repair and has been directly associated with postoperative pain, especially when tacks are used [12]. This is a well-recognized disadvantage of IPOM, which appears to be alleviated by minimal or absent fixation, as employed in the V-TAPP approach. Our results support this hypothesis, as V-TAPP was found to be associated with significantly less levels of pain 24 h postoperatively under identical analgesic regimens. The higher levels of postoperative pain are most likely the reason for longer hospitalization of IPOM cases. In our series, the in-hospital stay of V-TAPP patients was significantly shorter than those in the IPOM group. This finding might have been even more pronounced in healthcare systems outside of Germany, with a more outpatient-care oriented structure. Technical simplicity of IPOM is definitely one factor contributing to its widespread adoption. In contrast, V-TAPP is a more technically demanding procedure that requires a higher level of minimally invasive surgical skill to adequately develop the peritoneal flap, ensuring complete mesh coverage without any button-hole lesions, while avoiding entry into the retrorectus space [13]. We had one case of extensive peritoneal lesions that was converted from V-TAPP to IPOM. The technical complexity is reflected in operative time. In our series V-TAPP procedures were found to take approximately 30 min longer than IPOM. We advocate pre-dissection of the preperitoneal space by pneumodissection using a Veress needle, as this may facilitate more rapid establishment of the working space and peritoneal flap advancement, while potentially reducing peritoneal injury compared to direct peritoneal incision without prior extraperitoneal insufflation. To our knowledge, there is no trial directly evaluating the efficacy of this maneuver. Postoperative morbidity was found to be equally low in both groups. Seroma formation at 30 days after surgery was found to be slightly more for the V-TAPP group, without reaching statistical significance. One of the two seromas recorded underwent two punctions until it eventually resolved. At long-time follow-up both methods were found to perform equally good with regard to hernia recurrence. Of note, closure of the hernia defect was performed in both groups. The advantages of primary defect closure are well known in IPOM repairs [14]. Defect closure, along with the mesh placement, constitute the two most crucial aspects shared by both surgical approaches, that contribute to such a low hernia recurrence rate. Infection rate was also null, both perioperatively as well as at long-term follow up.

The observed advantages of V-TAPP align with the broader shift toward extraperitoneal mesh placement [7]. A wide palette of extraperitoneal advanced approaches exist, ranging from the extended total extraperitoneal approach (e-TEP), transabdominal retromuscular umbilical prosthetic repair (TARUP) to hybrid approaches such as Mini- or Less-Open Sublay repair and others. While all above mentioned approaches share the advantages that derive from the extraperitoneal mesh positioning, they remain technically challenging, especially when the surgeon finds themselves in the learning curve [15]. These techniques involve extensive retrorectus dissection or complex component separation, which may not be justified for smaller abdominal wall defects. Conversely, V-TAPP is a technique that produces operative trauma that is proportionate to the defect to be repaired, without the need for extensive dissection and/or dedicated material. Consequently, V-TAPP represents a highly accessible, low-complexity alternative specifically for small-to medium-sized primary umbilical hernias.

An obvious limitation of our study is its retrospective nature and lack of randomization. A cost analysis was not conducted in order to objectively demonstrate an economical advantage favoring the V-TAPP approach. Postoperative length of stay reflects national reimbursement characteristics in Germany, and the general tendency to hospitalize hernia repair cases. A further study limitation lies within the fact that the comparison remains based on two non-contemporaneous cohorts, Nevertheless, all patients received uniform institutional care throughout the study duration, which consistently utilized single-shot prophylaxis, standardized preoperative analgesia, and identical postoperative nutritional protocols. In addition, the relatively small sample sizes of both cohorts, combined with the low rate of postoperative complications, may limit adequate statistical power for analysis and conclusions. Randomized controlled trials with standardized surgical protocols and long-term follow-up are needed to reach solid conclusions.

## Conclusion

V-TAPP is a safe and efficient method for the treatment of primary umbilical hernia, associated with reduced postoperative pain and shorter hospital stays compared to IPOM, which nonetheless remains an acceptable alternative.

## Data Availability

The raw data supporting the conclusions of this article will be made available by the authors, without undue reservation.
